# Anæsthetics: Their Uses and Administration

**Published:** 1900-09

**Authors:** 


**Anæsthetics: their Uses and Administration.** By DUDLEY WILMOT BUXTON, M.D. Third Edition. Pp. xvi., 320. London: H. K. Lewis. 1900.

The third edition of this well-known work contains many important alterations and additions. Of especial interest are the chapters devoted to the administration of nitrous oxide gas, gas with air, and gas with oxygen. Mr. Buxton's statement that there is no advantage in using gas, or gas and air, for prolonged operations, will meet with general acceptance from anæsthetists who have given the method a trial. Not only may the patient suffer from asphyxial symptoms, but the operator is apt to be inconvenienced by the muscular spasms and incomplete relaxation. Whilst we are prepared to admit that gas and oxygen is one of the safest and easiest methods of anæsthetization, we have found that for general work the apparatus is too cumbrous and the expense too high. The technique of rectal etherization, and of the administration of ether and oxygen, are clearly described, and well merit more attention than has been paid to them up to the present time. We welcome, too, the chapter devoted to local anæsthesia by cocaine, and by the diluted mixtures of Schleich and others. There are a few clerical errors in the book; *e.g.*, "painful" on page 282, line 8, should read "painless." Mr. Dudley Buxton's book may be cordially recommended, both as a text-book and a work of reference upon anæsthetics.

